# Investigation and Assessment of AI’s Role in Nutrition—An Updated Narrative Review of the Evidence

**DOI:** 10.3390/nu17010190

**Published:** 2025-01-05

**Authors:** Hanin Kassem, Aneesha Abida Beevi, Sondos Basheer, Gadeer Lutfi, Leila Cheikh Ismail, Dimitrios Papandreou

**Affiliations:** 1 Department of Clinical Nutrition and Dietetics, College of Health Sciences, University of Sharjah, Sharjah P.O. Box 27272, United Arab Emirates; hkassem@sharjah.ac.ae (H.K.); u22200551@sharjah.ac.ae (A.A.B.); u23102357@sharjah.ac.ae (S.B.); u23103568@sharjah.ac.ae (G.L.); lcheikhismail@sharjah.ac.ae (L.C.I.); 2 Nuffield Department of Women’s & Reproductive Health, University of Oxford, Oxford OX1 2JD, UK

**Keywords:** nutrition, artificial intelligence, dietary assessment, personalized nutrition, machine learning

## Abstract

Background: Artificial Intelligence (AI) technologies are now essential as the agenda of nutrition research expands its scope to look at the intricate connection between food and health in both an individual and a community context. AI also helps in tracing and offering solutions in dietary assessment, personalized and clinical nutrition, as well as disease prediction and management, such as cardiovascular diseases, diabetes, cancer, and obesity. This review aims to investigate and assess the different applications and roles of AI in nutrition and research and understand its potential future impact. Methods: We used PubMed, Scopus, Web of Science, Google Scholar, and EBSCO databases for our search. Results: Our findings indicate that AI is reshaping the field of nutrition in ways that were previously unimaginable. By enhancing how we assess diets, customize nutrition plans, and manage complex health conditions, AI has become an essential tool. Technologies like machine learning models, wearable devices, and chatbot applications are revolutionizing the accuracy of dietary tracking, making it easier than ever to provide tailored solutions for individuals and communities. These innovations are proving invaluable in combating diet-related illnesses and encouraging healthier eating habits. One breakthrough has been in dietary assessment, where AI has significantly reduced errors that are common in traditional methods. Tools that use visual recognition, deep learning, and mobile applications have made it possible to analyze the nutrient content of meals with incredible precision. Conclusions: Moving forward, collaboration between tech developers, healthcare professionals, policymakers, and researchers will be essential. By focusing on high-quality data, addressing ethical challenges, and keeping user needs at the forefront, AI can truly revolutionize nutrition science. The potential is enormous. AI is set to make healthcare not only more effective and personalized but also more equitable and accessible for everyone.

## 1. Introduction

Although artificial intelligence (AI) has become famous recently, it has been a part of both practical applications and theoretical computer science for a long time. According to Russell and Norvig, “AI is the field that deals with the construction and analysis of procedures that have the properties of intelligence”. As many today consider AI as a science aiming to undertake activities that usually require human intelligence, its goals include performing tasks that require high-level cognitive functions, including visual perception, speech recognition, decision making, and general intelligence.

There are two types of AI: narrow (or weak) AI, which is focused on specific activities such as natural language processing or facial recognition, and broad (or strong) AI, which is more focused on developing general systems that can learn across various domains, embodying human terms of insight. With enormous potential, the AI field includes machine learning (ML), deep learning (DL), natural language processing (NLP), and computer vision (CV) [1].

Among AI’s key components is machine learning (ML); it assists systems in looking through large volumes of data to find patterns or connections that might not be immediately clear. Because of its ability to deal with non-linear and non-stationary issues, which occur more frequently in the nutrition and medicine fields, ML is particularly effective in those fields. For instance, ML algorithms can identify various diets and their relevance to the risk of chronic diseases, integrate data that predicts health outcomes based on genetic information, and automatically increase or decrease dietary recommendations depending on the amount of data gathered. This capacity for analysis and prediction makes ML a cornerstone of modern AI applications [1].

The use of AI tools in nutrition has found applications in identifying and even forecasting health concerns and analyzing nutritional databases, ensuring that the growth of this area is projected to thrive within the next decade. These barriers are broken down by employing AI processing capacity, which is able to integrate diet intake, genes, and biomarkers towards developing a personalized approach. In the field of medicine, AI based on ML technology has been helpful in the areas of diagnosing diseases, monitoring patients, and developing drugs. For instance, ML algorithms have made it possible to detect diabetes and cardiovascular diseases much earlier than before by utilizing non-linear relationships within the patient data, thus improving clinical decisions [1,2]. AI will help extend itself further in formulating targeted nutritional interventions for individual lifestyles based on clinical nutrition in general. It can help identify potential disease triggers, make appropriate clinical decisions, and improve the overall healthcare experience using personalized solutions [1,2]. AI-based tools can also record dietary data and develop appropriate meal strategies while assessing user achievement concerning improved health variables. In populations characterized by poor eating habits, AI-powered solutions can assist in mitigating such tendencies and offer useful dietary recommendations, which is of dire need given the report from the WHO on the causes of dietary disease [3].

Artificial intelligence technologies are now essential as the agenda of nutrition research expands its scope to look at the intricate connection between food and health in both an individual and a community context. AI also helps in tracing and offering solutions to illnesses such as cardiovascular diseases, diabetes, cancer, and obesity. There are also implementable AI devices that allow researchers to study the connection between nutrition and health outcomes in a way that would otherwise be difficult [2,4,5]. The United States is projected to take into use AI in healthcare systems within the next three years, where an estimated USD 150 billion is expected to be saved concerning operational costs, bringing about a more effective service provision in the health sector around the world, especially in areas with limited access to health care [6,7].

The purpose of this review is to provide a comprehensive analysis of the current applications of artificial intelligence (AI) in the field of nutrition, particularly in dietary assessment, personalized nutrition, disease management, and nutrition education. By systematically investigating these areas, this review aims to investigate and assess the different applications and roles of AI in nutrition and research and understand its potential future impact.

## 2. Materials and Methods

A comprehensive literature search was conducted across three databases: PubMed, Google Scholar, Scopus, Web of Science, and EBSCO. This search covered publications from 2003 to 2024 and included terms related to AI and nutrition, such as “artificial intelligence”, “machine learning”, “deep learning”, “dietary assessment”, “nutritional analysis”, “clinical nutrition”, “nutrition education”, and “personalized nutrition”. Boolean operators (AND, OR) were utilized to refine the search results. Additionally, extra records were identified by tracking the citations of the included studies.

## 3. Applications of AI in Nutrition

### 3.1. AI in Dietary Assessment and Tracking

#### Currently Implemented AI Tools

Dietary assessment is one of the most important steps in the nutrition care plan and the most concerning issue in recent nutrition research. Multiple traditional nutrition assessment tools are used by dietitians, but these methods are mostly dependent on memory, where they are subjected to human errors, and need a highly experienced and skilled interviewer to enable the collection of useful and reliable data.

The use of AI in dietary assessment may reduce this bias, as it exceeds the accuracy of humans, which could enhance dietary evaluations by optimizing efficiency and correcting both systematic and random inaccuracies that come with self-reported dietary intake data; additionally, it holds a potential as a means to address the pressing demand for precise and minimal-effort dietary evaluation [8].

Vision-based AI dietary assessment (VBDA) methods involve capturing images of meals as input and utilizing computer vision to automatically detect pertinent dietary details as the output. In the past, VBDA was performed through a sequential process, known as multi-stage dietary assessment [9], which was composed of three main steps, including image analysis, portion estimation, and nutrient derivation. In recent years, with the rapid development of deep learning, instead of training series of models to handle subtasks at different stages, the end-to-end deep learning solutions for dietary assessment are proposed to apply input data to a single network for direct nutrient derivation [9]. However, despite the important role of deep learning in dietary assessment, it can only perform a single nutrient assessment at one time [9], so multi-task image-based learning (MTL) has been developed to achieve a more holistic VBDA framework, which is usually executed through either soft or hard parameter sharing of the hidden layers [9].

Several new methods have been investigated based on advanced deep learning, including a face recognition method that was investigated in Morocco, which is based on food classification, portion size estimation, and segmentation, where image analysis was done through an object recognition camera where an image was taken before and after eating and a unique number was given for each meal; after that, an estimation of energy and nutrient composition will be accurately generated through the system [10].

In addition, a smartphone-based dietary assessment method, named go FOODTM, which is also based on the advanced (DL), has been recognized as a useful app in the estimation of micronutrients and energy content of food. It supports both images captured from two perspectives and stereo image pairs from smartphones that feature dual rear cameras as input [11]. Moreover, several mobile apps were developed for dietary tracking, including goFOOD^LITE^, which is a new version of goFood ^TM^ that is intended for logging the foods and drinks consumed by the user. The information gathered through this approach is suitable for looking back at past dietary habits. Consequently, this tool is beneficial for those who require a straightforward way to monitor their eating patterns, as well as for their dietitians or healthcare providers [11]. A recent study explored the feasibility of this app, which included 42 adults in Switzerland, by providing a feedback questionnaire regarding their satisfaction with its use. It concluded that 69% rated the logging and recording features as good or very good, while 83.3% found the app intuitive and easy to use. Regarding their willingness to use the app, 45.2% were neutral. Conversely, 52.4% expressed willingness to use the goFOOD^TM^ app for tracking their daily food intake, and 30 (71.4%) stated they would recommend it to friends [12].

Another common app in both dietary assessment and tracking is “Open Fit”, which can estimate and identify energy intake through short videos. Its validity and accuracy were addressed in a new pilot study that was performed on adults where they were asked to record 2 meals throughout the app. It found that portion size was not accurately estimated, as participants were asked to input the portion size manually, and an error in energy estimation in sugar, sweets, and beverages was noticed as errors for both automated and semiautomated estimates of energy were 10% and 314%, respectively [13].

Following this further, a critical review evaluated eighty (80) main food tracking applications (including My Fitness Pal, Meal Tracker, My Plate, Calorie Tip, etc.) and found that 3 out of 80 apps were able to recognize the food intake automatically and manually, those apps were Foodzilla! Nutrition Assistant, Food Diary, Recipe”, “Bitesnap: Photo Food Tracker & Calorie Counter”, and “Food Visor”. Additionally, most apps cannot estimate the food volume from food images and provide food recommendations according to the user’s needs [14].

### 3.2. AI in Personalized Nutrition and Diet Planning

#### Experimental AI Tools

AI is a progressively emerging tool that plays an important role in dietary management, where it provides creative approaches for personalized nutrition and health enhancement. Traditionally, dietary recommendations have been generalized, focusing on the requirements of the overall population instead of the unique individuals’ differences in metabolic health, lifestyle, or genetic composition. Nevertheless, this universal method frequently fails to address the individual nutritional requirements and personal health goals where the individuals will face a challenge in adherence to healthy diets in a lifelong manner due to multiple personal and environmental factors that are not addressed in this one-size-fits-all approach [15].

Therefore, the use of personalized approaches in nutrition support will enhance adherence to dietary recommendations and improve nutrition-health outcomes, as it is tailored according to the individual’s specific parameters, including genetic composition, medical condition, food preferences, personal habits, and specific health metrics like blood glucose levels [16].

Multiple AI techniques have been addressed in recent research including:

(a) Natural Language Processing (NLP): Where AI chatbots employ dialogues through natural language conversations with users through voice or text, which can enhance nutrition by providing customized diet recommendations for users. A recent study examined the efficacy of using an intelligent virtual assistant to guide users by answering their questions, offering portion size suggestions, and establishing goals for physical activity, aiming to motivate participants in a program designed to enhance their physical activity and dietary modifications. It showed improvements in physical activity, diet, and body composition [17], as participants increased their physical activity by an average of 109.8 min at week 12 compared to baseline. Mediterranean diet scores improved from a baseline average of 3.8 out of 14 to 9.6 at 12 weeks. After 12 weeks, participants experienced an average weight loss of 1.3 kg and a reduction of 2.1 cm in waist circumference [17].

(b) Machine learning (ML) is a branch of artificial intelligence (AI) that utilizes algorithms to perform tasks by analyzing vast amounts of data and identifying complex patterns within the data. It can generate personalized dietary recommendations tailored to an individual’s specific needs by integrating data from various sources, such as genetic profiles, biomarkers, dietary habits, and physiological responses. It also enables the identification of dietary patterns and their impact on health outcomes by facilitating the analysis of large-scale dietary surveys. Moreover, machine learning algorithms enhance food analysis and classification, streamlining the estimation of portion sizes and nutrient composition. In addition, this technology plays a crucial role in automating dietary adherence monitoring while offering valuable feedback to individuals striving to achieve specific dietary goals [18].

(c) Compared to traditional statistical methods, machine learning (ML)-based classification offers several benefits, such as reducing misclassification rates, improving generalizability, and simplifying experimental procedures. Conventional methods for measuring energy expenditure, like doubly labeled water, indirect calorimetry using breathing masks, or direct calorimetry, are often costly, complex, and impractical for everyday use. In contrast, systems that analyze accelerometer data, with or without additional physiological inputs, provide a practical, affordable, and accurate way to estimate energy expenditure in real-life settings [19].

(d) Deep Learning (DL): A subset of machine learning (ML). It is founded on artificial neural networks (ANNs), which are constructed based on synaptic connections found in the human brain; it identifies the unique features within the data and determines if any adjustment to the classification is necessary [20]. It has several advantages in terms of dietary improvement as it enhances the personalization, accuracy, and efficiency of the diet, which in turn leads to better adherence to the dietary recommendations and better health outcomes. In addition, it forecasts individual responses to various nutrients, helping to create tailored nutrition plans [21].

In a recent study, a correlation algorithm was used to measure the relationship between the biomarkers, BMI, and diet using simple deep learning networks in order to set a supportive system based on nutrition genomics for individualized nutrition and health outcome evaluation [22] Table 1 presents all AI techniques in personalized nutrition and diet planning.

Apps like ChatGPT: This is a language processing model that was released publicly in November 2022. It can address inquiries about nutrition and diet-related health issues, recommend nutritious meals, and offer recipes. Although ChatGPT has generated significant public enthusiasm, concerns regarding the accuracy and privacy of generated information are present. A recent study done to assess the accuracy and safety of dietary advice for users with food allergies through ChatGPT revealed that meals were prepared according to the recent dietary recommendations; in contrast, some unsafe meals (with food allergens) and energy miscalculations were identified [23]. Moreover, a comparative analysis was done between ChatGPT and the Food4Me algorithm, which concluded that ChatGPT recommendations were associated with minor or major errors where it could not create a link between macronutrient or micronutrient intakes with consumption of specific foods, so as a result it failed to provide advice regarding the necessary dietary changes [24]. Conversely, another recent study done to evaluate the diet plans produced by ChatGPT by comparing them to two weight-loss diet plans that are used in weight-management clinics found that AI-generated diets were matched with utilized diet plans in clinics [25].

Wearable and Mobile Sensors: Numerous digital and wearable methods have been suggested for monitoring nutrition, primarily focusing on calorie consumption and expenditure. A smart dining table capable of measuring food weight has been introduced to monitor total calorie consumption. Additionally, food intake and portion size monitoring have been proposed through a system that utilizes cameras to capture images of food, extract features, and identify meals (and their various components), portion sizes, and total calories by referencing Internet databases. Other technologies, such as microphones, piezoelectric sensors, and accelerometers, have been shown to track biting, chewing patterns, and swallowing frequencies, along with arm and wrist motions, to evaluate calorie intake through physical activity. Despite these significant advancements in digital nutrition sensors, there is a critical need for further chemical-based molecular data regarding bodily responses and metabolic profiles to provide meaningful nutrition guidance and dietary suggestions. Currently, a continuous glucose monitoring sensor is the only available wearable sensor. The integration of innovative sensor tools with advanced AI technology is anticipated to transform PN and thus optimal health outcomes [26].

### 3.3. Disease Prediction and Management

#### Forecasting and Treating Illnesses

Modern eating habits and industrialization have increased the prevalence of non-communicable diseases like diabetes, cardiovascular disease, and cancer. At the individual level, traditional, population-based dietary recommendations often fail to reduce chronic diseases. AI’s ability to analyze complex information from genetics, the microbiome, and environmental factors enables a personalized diet. The analysis highlights challenges such as defining healthy diets that consider individual situations, accurately evaluating the effectiveness of interventions, overcoming data biases, and ensuring model generalizability. Despite these challenges, AI-driven methods include determining dietary risk factors, identifying bioactive compounds that help control sickness, and optimizing nutrition for longevity and well-being [27].

Diseases like liver cirrhosis (LC), of which diet-related metabolic disorders are currently a major cause, are directly influenced by dietary habits. In the past, alcohol consumption and viral hepatitis were the most common causes of LC; however, a large percentage of cases are currently caused by metabolic dysfunction, which is frequently connected to poor dietary practices [28]. Customized dietary therapies have been demonstrated to help reduce the progression of liver disorders and diseases with comparable metabolic foundations.

RHMS collects health information in real time and monitors patients’ vital signs through IoMT devices. These platforms allow doctors to access continuous patient data, which is important for dietary and lifestyle changes, facilitating early interventions. For instance, wearable sensors monitor physiological data like blood glucose levels, physical activity, and heart rate. The research discusses how the use of predictive analytics and machine learning in RHMS enhances decision making and helps healthcare providers tailor interventions more effectively [29,30]. Moreover, by delivering accurate, real-time monitoring that better captures changes in health status, these systems help address the limitations of traditional self-reporting methods [31].

1. The RO-SmartAging System

Wearable and noninvasive sensors are used in this system, which is designed for elderly people to track vital health metrics like heart rate, physical activity, and even environmental elements like air quality. Real-time analysis of these data supports actions that encourage independence and healthy aging. Additionally, the technology communicates with caregivers, giving them access to alarms and status updates for the patient. As people age, diet-related problems, including osteoporosis or cardiovascular risks from dietary imbalances, can be effectively managed with this [32].

2. The NeuroPredict System

This platform, which was created to assist patients with neurodegenerative diseases, evaluates the effects of nutrition on cognitive health and estimates health paths using cutting-edge AI approaches. NeuroPredict can offer diet-based insights specific to cognitive health by gathering information from multiple sources, including cognitive tests such as the Mini-Mental State Examination (MMSE) and the Alzheimer’s Disease Assessment Scale-Cognitive (ADAS-Cog). The diet can affect mental decline in people with conditions like dementia or Alzheimer’s; this platform highlights the significance of nutrition in preserving cognitive function [33,34,35].

3. The HepatoConect Mechanism

Patients with liver cirrhosis are the target of this system. HepatoConect tracks important health metrics and dietary compliance using wearable technology and IoMT. By sending notifications if health parameters deviate from typical ranges, the system’s real-time monitoring allows for prompt dietary or pharmaceutical modifications. In addition, it monitors symptoms like lethargy or jaundice, which are typical in people with liver disease, and offers dietary advice to better control these disorders [36].

### 3.4. Key Benefits of Smart Diet Management Solutions

(a) Extensive and Adaptable Monitoring

By integrating information from several sources, such as lifestyle and environmental factors, each RHMS offers a thorough health overview to guarantee a full picture of a patient’s health. With this method, medical professionals can evaluate patient compliance, tailor food recommendations, and promptly modify treatment. These systems provide a highly customized strategy that enhances patient outcomes by integrating vitals, exercise, and food data [36].

(b) Accuracy and Data-Informed Decision Making

RHMS technologies offer accurate insights and support for preventative care through wearable sensors and AI-driven predictive analytics. RHMS continually tracks health data, enabling dynamic decision making based on real-time information, in contrast to traditional techniques that depend on recurring check-ups. For the management of diet-related disorders, which frequently call for regular dietary and lifestyle changes, this proactive approach is crucial [37,38].

(c) Connectivity to Medical Systems

Effective disease treatment depends on improved communication between patients, caregivers, and physicians, which RHMS fosters through the creation of a connected ecosystem. RHMS solutions enable healthcare providers to stay up to date on a patient’s condition and react to changes in health more rapidly by enabling remote consultations and real-time data sharing. For diet-related conditions that need constant observation and modification, this collaborative care strategy is especially helpful [39].

### 3.5. Machine Learning Models

According to the study, four essential modules—personalized meal suggestions, medication reminders, activity tracking, and an instructional chatbot—are integrated to create a comprehensive machine learning-based diabetes management system that meets clinical needs [40]. To provide a highly customized management experience, the system design integrates an activity and medication reminder module, an educational module with food identification, and a diabetes intelligent meal recommender.

The Diabetes Intelligent Meal Recommender uses the Harris–Benedict equation to determine calorie requirements based on food nutritional information from sites such as MyFitnessPal and patient health information (age, activity level). The recommender provides meal recommendations that are tailored to the patient’s dietary requirements using a K-nearest neighbor (KNN) algorithm [41].

With the help of Microsoft QnA Maker, the educational module includes a Q&A chatbot that uses natural language processing and a food recognition model based on Google’s TensorFlow to answer patient questions about managing their diabetes. This technique helps with dietary compliance and making educated meal choices by recognizing regional cuisine from photos [42].

In addition to supporting adherence, the activity and medication reminder module offers doctors comprehensive glucose logs and medication data through geolocation-based activity tracking and medication notifications [40].

Testing revealed encouraging outcomes for the system’s implementation, which was built on the Ionic framework for cross-platform capability. The chatbot correctly answered questions about diabetes, the medication reminder was made to ensure that people took their medications on time, and the meal recommender classified meals with an accuracy of 93–99%. Although improvements in low-quality image handling and increased calorie monitoring capabilities are advised, this integrated approach demonstrates the potential of machine learning for efficient and accessible diabetic self-management [43]. This solution combines evidence-based dietary, educational, and activity modules with user-friendly technology, demonstrating how sophisticated, AI-driven frameworks can promote better diabetes treatment.

The following Table 2 provides an overview of key AI techniques, their applications in nutrition, implementation status, and limitations.

## 4. AI in Nutrition Practice and Research

Artificial intelligence (AI) is rapidly advancing in healthcare and has the potential to transform clinical nutrition (Table 3). It can improve patient outcomes and support healthcare professionals with personalized, evidence-based approaches. However, its integration must be carefully monitored to ensure safety [44]. Patients can use AI techniques like wearable sensors to self-monitor variables, enabling personalized management of nutrition-related issues [45].

AI enhances parenteral and enteral nutrition formulas by efficiently adjusting plans to meet individual patient needs and monitoring health to schedule follow-ups for abnormal results [44]. A machine learning model can predict the likelihood and severity of adverse drug reactions based on factors like circulatory system diseases and parenteral nutrition in critically ill neonates [45].

AI-enabled devices improve dietary assessments by analyzing acoustic signals, jaw movements, and images of eating. Deep learning (DL) allows for rapid muscle mass evaluations through CT imaging, predicts early enteral nutrition needs for ICU patients, and verifies nasogastric tube (NGT) placement with chest X-rays. Machine learning (ML) tools assess the risk of refeeding syndrome, while wearable devices monitor hydration and detect infections in patients on home parenteral support [44].

A machine learning technique using extreme gradient boosting can predict refeeding hypophosphatemia in ICU patients resuming enteral or parenteral nutrition after prolonged fasting. Additionally, artificial intelligence is used to predict feeding intolerance in septic ICU patients based on nutrient type, feeding method, and health conditions. ML models analyze data from ICU patients to predict early enteral nutrition (EN) failure, which is defined as inadequate delivery by the third day. This helps clinicians identify high-risk patients who could benefit from early nutrition intervention [46]. The eTRIP app is an AI-driven program focused on weight loss through healthy eating. It is based on a modified temporal self-regulation theory and behavioral change taxonomy. The development of the eTRIP app involved two phases: (a) creating an AI-assisted self-monitoring system and (b) developing an AI-assisted behavioral nudging system [47]. XGBoost, an AI-based model, analyzes digitalized medical data to predict a patient’s 1-year HbA1c change and helps nutritionists make informed decisions about appropriate nutritional interventions [48]. AI-integrated smart toilets can monitor bowel movements, detect blood in the stool, and measure stool or stoma output, providing valuable data for managing hydration and fluid balance [44].

### 4.1. Support Clinical Decision Making

Artificial intelligence (AI) is used in decision support systems (DSS) to assist health professionals in complex decision-making processes regarding treatment options or diagnoses for malnutrition or cancer treatment [45].

Explainable artificial intelligence (XAI) and fuzzy logic-based reasoning are used in clinical decision support systems (CDSS) to assess nutrition-related issues in geriatric patients, such as malnutrition, oropharyngeal dysphagia, dehydration, and eating disorders in those with dementia. A prototype CDSS was developed and tested on geriatric patients, demonstrating high accuracy compared to expert evaluations [49].

### 4.2. Malnutrition Detection and Monitoring

AI improves malnutrition screening through the MUST-Plus model, which uses electronic health record (EHR) data and machine learning to accurately predict malnutrition risk by analyzing clinical assessments, physiological data, and lab results [46]. Additionally, AI enables early diagnosis and personalized care by integrating various health data [50]. The convolutional neural network (CNN) algorithm efficiently identifies children at risk of malnutrition and provides personalized intervention recommendations [51].

A machine learning model for identifying malnutrition in cancer patients shows moderate agreement with established tools like the Patient-Generated Subjective Global Assessment. Integrating advanced AI-assisted radiological imaging into malnutrition screening can enhance the detection of sarcopenia and accurate risk identification [45]. AI offers scalable solutions to tackle malnutrition using modeling strategies such as supervised learning for classification and unsupervised learning for pattern recognition. Deep learning aids in predictions, while cognitive learning supports language processing. Metamodeling-based sensitivity analysis helps identify actionable patterns in clinical parameters [50].

### 4.3. Nutrition Education

Effective communication skills are crucial for nutrition and dietetics practitioners. They should focus on building rapport, paraphrasing, demonstrating empathy, employing clinical reasoning, and practicing active listening. Improving these skills enhances patient satisfaction and clinical outcomes. Virtual simulated patients (VSPs) offer a new, more accessible approach to communication training in dietetics education. The use of virtual simulated patients (VSPs) in health professions education is increasing, with evidence indicating that they can enhance both communication skills and clinical reasoning. Developing VSPs has traditionally been resource-intensive, but the arrival of large language models like ChatGPT offers new opportunities for integrating artificial intelligence into VSP applications [52].

ATLAS (authentic teaching and learning application simulation), an innovative educational platform that integrates voice and chat features, utilizing advanced large language models, artificial intelligence, and educator-created patient personas to simulate real-world human-simulated patients (HSPs), which is designed to improve communication skills in dietetics education by providing students with authentic learning experiences, multiple attempts, and instant personalized feedback [52]. Dietitians and nutrition professionals can use AI to analyze large datasets and provide customized educational content for vulnerable groups. This enhances the efficiency of nutrition education and promotes healthier dietary habits [53]. AI prediction systems have great potential to create a personalized health education system, help shared decision making, and improve the effectiveness of diabetes nutrition counselling and health education [48].

AI chatbots, like ChatGPT, can provide instant, scientifically supported nutritional advice. These bots are trained on extensive datasets from texts, books, articles, and other online sources. As a result, they can clarify common myths, offer dietary recommendations, and explain complex nutrition topics in real time [54]. Furthermore, educational institutions have started utilizing ChatGPT-powered chatbots to reinforce their health and nutrition [54] (Table 4).

### 4.4. Clinical Nutrition Research

AI techniques are used in projects designed to create tools that support dietary activities [1].

GoCARB is a smartphone app that uses computer vision to estimate the carbohydrate content in plated meals, providing accuracy similar to professional dietitians for managing carbohydrate intake in patients with type 1 diabetes [55]. Database matching algorithms were developed to link foods in the Nutrition Coordinating Center (NCC) database with those in the ASA24, using either nutrient data alone or a combination of nutrients and food descriptions. These computational methods effectively estimate an NCC-exclusive nutrient for foods reported in ASA24 based on the combination of nutrients and food descriptions [56].

A rapid automatic bite detection algorithm (RABID) uses video skeletal features to detect and analyze eating behaviors, with a high level of agreement [57]. The machine learning model achieved a 91.2% accuracy in identifying mild dehydration by analyzing data of autonomic indicators like electrodermal activity (EDA) and pulse rate variability (PRV) during the Stroop test [58]. An OWL-based knowledge-based system (KBS) is developed to recommend appropriate food servings for chronic kidney disease patients, demonstrating accurate reasoning and the flexibility to expand its knowledge base [59]. A hybrid food recommendation method was developed, utilizing chronic disease-based clustering and a nutrition knowledge base. Using the k-means algorithm, food products are categorized based on nutrient data, creating a knowledge base incorporating food preferences [60]. MIKAS (menu information and knowledge acquisition system) is an AI prototype for menu planning using an incremental knowledge acquisition system. It collects expert explanations of their decisions to enhance its knowledge base, enabling automatic menu construction in the future [61].

“Nutri-Educ” is a software tool that uses fuzzy arithmetic to balance meals based on a patient’s energy needs. The software uses heuristic search algorithms to improve the nutritional quality of an initial meal, turning it into a well-balanced option [62]. “NutriNet” is a mobile app designed for dietary assessment using food image recognition in patients with Parkinson’s disease, utilizing a deep convolutional neural network tool [63].

Artificial intelligence is used to diagnose and predict the risk of chronic diseases [1]. AI techniques like the k-nearest neighbors algorithm and random forest decision trees effectively estimate health risks based on dietary and supplementation patterns. They assess 10-year cardiometabolic risk with machine learning and outperform linear regression in health score classification [64]. A machine learning model was developed to predict individual variability in postprandial metabolic responses to food intake [65]. A breast cancer prediction model was developed using an artificial neural network (ANN) to assess the impact of micronutrients like folate and B12 on cancer risk, explaining 94.2% of the variability in predictions [66]. Machine learning validation techniques, such as ensemble methods, generalized regression prediction, elastic net, and leave-one-out cross-validation, were used to validate how healthy eating (measured by the HEI index) influences cancer prognosis [67] (Table 4).

## 5. Challenges and Limitations

### 5.1. Data Privacy and Ethical Concerns

The incorporation of AI into personalized nutrition offers significant opportunities as well as notable challenges, particularly regarding data privacy and ethical standards. AI-driven systems rely on sensitive health information, including electronic health records (EHRs), genetic information, and dietary habits, to deliver tailored nutritional advice. Although this information is crucial for producing accurate recommendations, it also creates risks related to data breaches and possible misuse. For instance, incidents of unauthorized data sharing in collaborations between public and private entities, like the NHS-Google partnership, illustrate the dangers associated with privacy protections. Such cases underscore the essential need for robust data governance frameworks, clear standards, and a commitment to safeguarding patient data to maintain public confidence and ethical integrity [68,69].

The growing ability of advanced algorithms to re-identify anonymized data raises important ethical issues in this area. This ability raises concerns about the effectiveness of existing anonymization methods, increasing the likelihood that confidential data may be exploited for activities without permission, such as targeted advertising or discrimination. In response to these challenges, blockchain technology has emerged as an alternative solution, offering decentralized and unchangeable data storage that enhances security and dependability. By utilizing blockchain, AI systems can secure data integrity and promote increased trust in AI applications. Furthermore, dynamic consent frameworks give individuals the ability to retain control over their personal information by allowing them to change or revoke consent as necessary, ensuring compliance with ethical standards in evolving AI environments [70].

Transparency poses a significant challenge within AI systems, particularly those described as “black boxes”. The unclear workings of these systems lead to concerns about accountability and the ethical management of patient information. Both users and healthcare providers frequently encounter challenges due to the ambiguity surrounding AI-generated decisions, which can foster distrust among patients and healthcare providers [44]. Regulatory measures such as the General Data Protection Regulation (GDPR) and the Health Insurance Portability and Accountability Act (HIPAA) are crucial in addressing these issues [71]. These regulations mandate strong encryption techniques, safe data storage methods, and consent mechanisms centered on patients. These frameworks emphasize the ethical handling of sensitive data, including genomic and nutritional details, to ensure that AI systems meet societal expectations while complying with legal and regulatory obligations [72].

Moreover, tools driven by AI, such as dietary tracking applications, gather a variety of personal information, ranging from anthropometric measures to psychological health indicators, which raises privacy concerns. Although these systems provide advantages in terms of efficiency, cost savings, and convenience, their reliance on sensitive information necessitates strict ethical oversight. To mitigate the risk of misuse and foster user confidence, sophisticated anonymization methods and secure data-sharing practices must be supported by robust accountability mechanisms. Addressing these issues is essential for encouraging the ethical application of AI in personalized nutrition, all while preserving user autonomy and trust [73].

### 5.2. Data Quality and Bias

The quality and diversity of datasets used to train AI models are critical for assuring personalized nutritional recommendations’ fairness, precision, and reliability. Unfortunately, existing datasets frequently lack adequate demographic representation, resulting in biased AI outputs that disproportionately affect marginalized or underrepresented populations. For example, data skewed toward Western populations may overlook cultural dietary patterns or economic constraints in other regions, leading to impractical or irrelevant recommendations [74]. Such biases not only undermine the credibility of AI systems but also worsen existing health disparities by neglecting the unique nutritional needs of different populations.

Integrating culturally varied datasets is crucial for mitigating biases. This guarantees that algorithms make equitable and inclusive recommendations for various populations. Collaborations with local researchers and institutions can increase the relevance of AI solutions by tailoring them to region-specific dietary behaviors and nutritional requirements [75].

Self-reported data, which are a prevalent and easy-to-obtain source for AI models, complicate the matter further by introducing errors stemming from recall bias, deliberate misrepresentation, and varying cultural interpretations of food. For example, dietary habits in Southeast Asia often feature local dishes that AI models trained mainly on Western data might not recognize. This disparity underscores the importance of regionally tailored AI tools, like eTRIP, that incorporate local food identification features. Although these approaches exhibit the potential of context-focused AI applications, their ability to scale is frequently obstructed by the absence of high-quality, localized datasets [47]. Similarly, dependence on pre-existing datasets like “Food-101” reveals the difficulties in applying generalizations across a variety of dietary practices without deliberately integrating culturally specific information [76].

### 5.3. Reliability and Model Accuracy

Ensuring the reliability and precision of AI-generated nutritional advice is a complicated task influenced by multiple factors, including data integrity, model structure, and the inherently changing nature of human health. A significant number of AI systems rely on self-reported dietary information, which is naturally subject to inaccuracies such as recall bias, deliberate underreporting, and cultural differences in food classification and consumption. These inaccuracies undermine the accuracy of AI-generated recommendations, often leading to less-than-ideal or even inappropriate dietary advice [68,77].

For instance, when tasked with creating meal plans for individuals with chronic conditions such as diabetes, AI systems often fall short in meeting specific nutritional needs, including accurate carbohydrate counting or sodium limits essential for managing hypertension [78,79]. These limitations are especially concerning for patients dealing with multiple health issues, where it is necessary to balance conflicting dietary requirements. Such difficulties highlight the importance of a human vital role in improving AI-generated outputs, particularly in complex scenarios that require a deeper understanding and clinical expertise. Moreover, the opaque nature of many AI models complicates their internal decision-making processes, making it challenging for users and healthcare providers to evaluate the credibility and relevance of their recommendations. This lack of transparency can undermine trust and hinder the integration of AI systems into important healthcare applications [70].

AI systems are required to navigate the inherent complexities of human nutrition, shaped by interactions between genes and diet, as well as variations in individual metabolic responses. These complicated elements demand ongoing updates to models and practical testing in real-world scenarios to guarantee that insights derived from AI stay scientifically valid and relevant [44,80].

## 6. Strengths and Limitations

This review article provides a well-structured and thorough analysis of the role of artificial intelligence (AI) in nutrition, covering various applications and methodologies. The strengths of this review lie in its comprehensive coverage, from AI in dietary assessment and personalized nutrition to disease prediction and management. The inclusion of a wide range of studies from reputable sources spanning two decades (2003–2024) enhances the reliability of the findings. Moreover, the review incorporates real-world case studies, such as the use of AI-driven apps like goFOODTM and Open-fit, which provide concrete examples of AI’s practical applications in nutrition. The clear methodology for data collection and inclusion criteria for the literature review adds transparency to the process, ensuring that the conclusions are based on solid evidence. The review also thoughtfully addresses the potential challenges, including ethical concerns and data privacy, providing a balanced view of both the opportunities and obstacles in integrating AI with nutrition. Despite its strengths, the review has a few limitations. One significant limitation is the broad scope of the review, which, while beneficial in covering various aspects of AI in nutrition, can result in a lack of depth in certain areas. For instance, the challenges of implementing AI tools in low-resource settings or culturally diverse populations are not explored sufficiently. Additionally, while the review discusses ethical concerns and data privacy issues, it does not provide in-depth solutions or strategies to address these challenges, leaving a gap in practical recommendations. Furthermore, the reliance on secondary data and studies with varying methodologies may introduce inconsistencies or biases, which could affect the robustness of the conclusions. The review also lacks a critical examination of the limitations of the reviewed studies themselves, such as potential conflicts of interest or methodological weaknesses. Lastly, while the review highlights the potential of AI, it could benefit from more discussion on the scalability and real-world effectiveness of AI tools, particularly in diverse healthcare settings.

## 7. Future Directions

### 7.1. Advancements in AI for Personalized Nutrition

The emergence of adaptive and predictive AI models is transforming the field of personalized nutrition by facilitating tailored dietary recommendations that cater to individual health requirements through the incorporation of real-time data. These models dynamically adjust to a person’s specific health conditions, lifestyle habits, and preferences, providing a high level of personalization to effectively enhance nutritional strategies [74]. By utilizing vast datasets and machine learning algorithms, these systems optimize nutritional strategies, anticipating and modifying dietary plans based on the changing health needs of individuals, delivering data-informed solutions that are tailored to specific individuals [81]. Factors such as genetics, microbiome composition, and cultural food preferences are taken into account in these AI-driven systems, paving the way for precision nutrition interventions designed to enhance health outcomes across various populations [82,83].

One of the most significant advancements in this field is the incorporation of wearable technologies and real-time feedback systems, which improve the precision of dietary pattern assessments, nutrient intake evaluations, and food image identification. These tools guarantee that nutritional guidance is not only tailored to the individual but also accurate and practical, meeting a vital demand for immediate adaptability [84]. Furthermore, these systems are broadening the horizons of nutrition science by tackling pressing global issues such as malnutrition and the management of diet-related illnesses. They serve a wide range of demographics by offering culturally sensitive and inclusive solutions, ensuring their effectiveness across various settings [77,85]. Looking forward, the future of AI in personalized nutrition is centered on the ongoing development of resilient, flexible systems that can progress alongside personal health measurements to deliver meaningful and individualized support [86,87].

### 7.2. Ethical Framework and Regulatory Standards

The integration of artificial intelligence into the fields of healthcare and nutrition highlights the urgent requirement for standardized ethical guidelines and regulatory standards to guarantee its secure, equitable, and responsible usage. These guidelines are essential for tackling significant issues such as biases present in AI algorithms, protecting user data privacy, and maintaining transparency in AI decision-making procedures [74,81]. Formulating these guidelines is crucial not only for fostering trust in AI technologies but also for safeguarding sensitive data and ensuring fair access to AI-driven solutions across different demographic and socioeconomic groups [82].

To avoid potential abuse and reduce bias, policymakers, technologists, healthcare professionals, and other stakeholders must collaborate. These initiatives should aim to establish comprehensive global standards that emphasize inclusivity by integrating diverse data sources, ensuring that AI systems operate fairly for all population groups [84,88]. Additionally, regulatory frameworks need to be flexible and able to adapt alongside technological progress, maintaining a balance between nurturing innovation and ensuring ethical obligations [44,68]. In significant applications, such as dietary interventions for the prevention and management of diseases, ethical supervision is essential to avoid harm and support the responsible use of these advanced technologies [87,89].

Additionally, ethical frameworks should prioritize inclusivity and accountability by promoting interdisciplinary collaboration, conducting regular audits, and establishing transparent governance methods. These actions will protect individual rights while also facilitating the sustainable and fair integration of AI technologies in the fields of nutrition and healthcare [86,90]. By integrating ethical considerations into the foundation of AI development, stakeholders can guarantee that these transformative tools provide significant advantages without violating ethical principles.

Successfully integrating AI in personalized nutrition necessitates a thorough approach to address these challenges. This requires collaboration among different stakeholders, such as lawmakers, technology innovators, and healthcare professionals, to create a cohesive framework that harmonizes innovation with ethical standards. By prioritizing user autonomy, improving transparency, and establishing strong protections, the industry can unlock its full potential while safeguarding individuals’ rights and building trust. Addressing privacy and ethical concerns is not just a legal requirement but also an essential foundation for promoting sustainable progress in AI-based nutrition solutions.

## 8. Conclusions

To sum up, AI is reshaping the field of nutrition in ways that were previously unimaginable. By enhancing how we assess diets, customize nutrition plans, and manage complex health conditions, AI has become an essential tool. Technologies like machine learning models, wearable devices, and chatbot applications are revolutionizing the accuracy of dietary tracking, making it easier than ever to provide tailored solutions for individuals and communities. These innovations are proving invaluable in combating diet-related illnesses and encouraging healthier eating habits. One breakthrough has been in dietary assessment, where AI has significantly reduced errors that are common in traditional methods. Tools that use visual recognition, deep learning, and mobile applications have made it possible to analyze the nutrient content of meals with incredible precision. At the same time, personalized nutrition has moved away from generalized recommendations to a more targeted approach, taking into account factors like a person’s genetics, metabolism, and lifestyle. This shift has helped individuals stick to healthier diets more consistently and achieve better health outcomes over the long term.

AI is also playing a critical role in predicting and managing chronic diseases such as diabetes, heart disease, and obesity. With wearable devices and real-time monitoring, remote health systems are making it easier to track vital signs and intervene early when issues arise. These tools are especially valuable in underserved areas, where access to traditional healthcare can be limited. By providing continuous care and immediate feedback, AI is bridging gaps in healthcare and improving patient outcomes worldwide.

However, using AI in nutrition is not without its challenges. The quality of data and the biases that can arise from it are key concerns. Many datasets lack diversity, which can result in recommendations that are less relevant for certain populations. Privacy is another pressing issue, as these systems often rely on sensitive personal information. Building trust requires making AI systems more transparent and ensuring they meet strict ethical and legal standards. Regulations like GDPR and HIPAA are steps in the right direction, but ongoing effort is needed to uphold these principles.

Moving forward, collaboration between tech developers, healthcare professionals, policymakers, and researchers will be essential. By focusing on high-quality data, addressing ethical challenges, and keeping user needs at the forefront, AI can truly revolutionize nutrition science. The potential is enormous—AI is set to make healthcare not only more effective and personalized but also more equitable and accessible for everyone.

## Figures and Tables

**Table 1:** AI techniques in personalized nutrition and diet planning.

Author and Year	AI Technique	Description
Maher et al., 2020 [17]	Natural language processing (NLP)	AI chatbots provide personalized dietary recommendations through natural language conversations. They guide users with portion size suggestions, physical activity goals, and dietary modifications.
Varshney et al., 2023 [18]	Machine learning (ML)	Uses data to generate personalized nutrition recommendations, identify dietary patterns and monitor food intake and nutrients composition
Abdullah et al., 2022 [21]	Deep learning (DL)	Based on artificial neural networks, it identifies unique features within datasets And helps in prediction of the nutrients relationship to humans, which will aid in creating individulaized diets
Niszczota and Rybicka, 2023 [23]	Apps like ChatGPT (limited implementation)	ChatGPT was evaluated for generating dietary recommendations. Meals created by ChatGPT were aligned with dietary guidelines, while inaccuracies in energy calculation and food allergens detection were observed
Agne and Gedrich, 2024 [24]		A comparison of ChatGPT’s recommendations with the Food4Me algorithm revealed errors in linking macronutrient and micronutrient intakes with specific foods
Kim et al., 2024 [25]		A study comparing ChatGPT-generated diet plans to weight-loss diet plans used in clinic settings found that AI-generated diets closely matched the utilized clinical plans
Sempionatto et al., 2021 [26]	Wearable and mobile sensors (implemented)	Wearable sensors and mobile systems monitor nutrition by tracking calorie intake, food weight, portion size and eating behavior. Technologies like microphones, cameras, and accelerometers, which evaluate the biting, chewing and swallowing patterns, can provide valuable data for personalized nutrition

**Table 2:** Summary of AI techniques, applications, implementation status, and limitations in nutrition.

AI Technique	Applications	Implementation Status	Limitations
Machine learning (ML)	Personalized nutrition, dietary pattern recognition	Partially implemented	Bias in training data
Deep learning (DL)	Nutrient composition estimation, image-based food tracking	Experimental phase	Requires large datasets
Natural language processing (NLP)	AI chatbots for dietary advice	Partially implemented	Accuracy of advice
Wearable sensors	Calorie tracking, real-time monitoring	Partially implemented	Limited availability of continuous glucose monitors
AI-Powered apps	Diet tracking and recommendations	Partially implemented	Accuracy and privacy concerns

**Table 3:** Application of AI in clinical nutrition.

Author and Year	AI Technique	Description
Bond et al., 2023 [44]	Deep learning (DL)	Deep learning (DL) allows for rapid muscle mass evaluations through CT imaging, (currently implemented in research settings and some clinical centers) predicts early enteral nutrition needs for ICU patients, (still requires more large-scale validation studies before widespread clinical adoption), and verifies nasogastric tube (NGT) placement with chest X-rays (being actively used in many hospitals)
	Machine learning (ML)	Assess the risk of refeeding syndrome, (currently experimental/research phase) while wearable devices monitor hydration (partially implemented), and detect infections in patients on home parenteral support (primarily experimental)
	AI-integrated smart toilets	Monitor bowel movements, detect blood in the stool, and measure stool or stoma output, providing valuable data for managing hydration and fluid balance. (Primarily experimental/prototype phase)
Janssen et al., 2024 [45]	Machine learning (ML)	Predict the likelihood and severity of adverse drug reactions based on factors like circulatory system diseases and parenteral nutrition in critically ill neonates. (Partially implemented in specialized centers)
	Machine learning (ML)	Identifying malnutrition in cancer patients shows moderate agreement with established tools like the Patient-Generated Subjective Global Assessment. (Currently in transitional phase between experimental and implemented)
	Advanced AI-assisted radiological imaging	Integrating malnutrition screening can enhance the detection of sarcopenia and accurate risk identification. (Partially implemented in clinical settings)
Kittrell et al., 2024 [46]	Extreme gradient boosting	Predict refeeding hypophosphatemia in ICU patients resuming enteral or parenteral nutrition after prolonged fasting. (Primarily experimental/research phase)
	Deep learning (DL) and machine learning (ML)	Predict feeding intolerance in septic ICU patients based on nutrient type, feeding method, and health conditions. (Primarily experimental phase)
	MUST-Plus model	Uses electronic health record (EHR) data and machine learning to accurately predict malnutrition risk by analyzing clinical assessments, physiological data, and lab results. (Partially implemented in selected healthcare systems)
Jocelyn Chew et al., 2024 [47]	eTRIP app	Supports weight loss with self-monitoring, AI-assisted behavioural nudging system. (Experimental phase)
Liu et al., 2023 [48]	XGBoost	Predict a patient’s 1-year HbA1c change and help nutritionists make informed decisions about appropriate nutritional interventions. (Research/validation phase)
Petrauskas et al., 2021 [49]	Explainable AI (XAI) and fuzzy logic-based reasoning	Used in clinical decision support systems (CDSS) to assess nutrition-related issues in geriatric patients, such as malnutrition, oropharyngeal dysphagia, dehydration, and eating disorders in those with dementia. (Limited clinical implementation)
	A prototype CDSS	Assesses malnutrition and related issues in geriatric patients with high accuracy compared to expert evaluations. (Experimental/prototype phase)
Sharma et al., 2020 [50]	Supervised learning Unsupervised learning Deep learning Cognitive learning Metamodeling-based sensitivity analysis	AI offers scalable solutions to tackle malnutrition using modeling strategies for classification, pattern recognition, predictions, language processing, and identifying actionable patterns in clinical parameters. (Full integration varies by institution)
Bhakti Vichave et al., 2023 [51]	Convolutional neural network (CNN)	Identifies children at risk of malnutrition and provides personalized intervention recommendations. (Experimental phase)

**Table 4:** Application of AI in nutrition education and clinical nutrition research.

Author and Year	AI Technique	Description
Barker et al., 2024 [52]	ATLAS platform	Combines voice, chat, and educator-created patient personas. Simulates Human Simulated Patients (HSPs) for real-world communication practice. It enhances dietetics education and improves communication skills. (Partially implemented in select dietetics programs)
Kalyoncu Atasoy et al., 2024 [53]	AI techniques	Analyze large datasets and provide customized educational content for vulnerable groups. This enhances the efficiency of nutrition education and promotes healthier dietary habits. (Partially implemented)
Liu et al., 2023 [48]	AI prediction system	Create a personalized health education system, help shared decision making, and improve the effectiveness of diabetes nutrition counselling and health education. (Experimental phase)
Arslan, 2024 [54]	AI chatbots (ChatGPT)	Can provide instant, scientifically supported nutritional advice and can clarify common myths, offer dietary recommendations, and explain complex nutrition topics in real time. (Partially implemented through various platforms)
Vasiloglou et al., 2018 [55]	GoCARB (smartphone app)	Estimate the carbohydrate content in plated meals, providing accuracy similar to professional dietitians for managing carbohydrate intake in patients with type 1 diabetes. (Partially implemented)
Chin et al., 2019 [56]	Database matching algorithms	Link foods in the Nutrition Coordinating Center (NCC) database with those in the ASA24, using either nutrient data alone or a combination of nutrients and food descriptions. These computational methods, effectively estimate an NCC-exclusive nutrient for foods reported in ASA24 based on the combination of nutrients and food descriptions. (Partially implemented in research settings)
Konstantinidis et al., 2020 [57]	RABID algorithm	Uses video skeletal features to detect and analyze eating behaviors, with a high level of agreement. (Highly experimental phase)
Posada-Quintero et al., 2020 [58]	Machine learning	Have accuracy in identifying mild dehydration by analyzing data of autonomic indicators like electrodermal activity (EDA) and pulse rate variability (PRV) during the Stroop test. (Experimental phase)
Chi et al., 2015 [59]	OWL-based knowledge system	Developed to recommend appropriate food servings for chronic kidney disease patients, demonstrating accurate reasoning and the flexibility to expand its knowledge base. (Limited clinical implementation)
Baek et al., 2019 [60]	Hybrid food recommendation method	Utilizing chronic disease-based clustering and a nutrition knowledge base. Using the k-means algorithm, food products are categorized based on nutrient data, creating a knowledge base incorporating food preferences. (Experimental phase)
Salam Khan and Hoffmann, 2003 [61]	MIKAS (AI prototype)	Plans menu using an incremental knowledge acquisition system. It collects expert explanations of their decisions to enhance its knowledge base, enabling automatic menu construction in the future. (Not yet clinically implemented)
Buisson, 2008 [62]	Nutri-Educ(software uses fuzzy arithmetic)	Balance meals based on a patient’s energy needs. The software uses heuristic search algorithms to improve the nutritional quality of an initial meal, turning it into a well-balanced option. (Partially implemented)
Mezgec and Seljak, 2017 [63]	NutriNet (mobile app)	Designed for dietary assessment using food image recognition in patients with Parkinson’s disease, utilizing a deep convolutional neural network tool. (Experimental phase)
Panaretos et al., 2018 [64]	AI techniques	K-nearest neighbors algorithm and random forest decision trees estimate health risks based on dietary and supplementation patterns effectively. They assess 10-year cardiometabolic risk, with machine learning and outperform linear regression in health score classification. (Partially implemented in research settings)
Berry et al., 2020 [65]	Machine learning	Predict individual variability in postprandial metabolic responses to food intake. (The field is rapidly evolving, with some applications already commercially available while more advanced applications are still being developed and validated through research.)
Naushad et al., 2016 [66]	Artificial neural network (ANN)	A breast cancer prediction model, assesses the impact of micronutrients like folate and B12 on cancer risk, explaining 94.2% of the variability in predictions. (Currently in Use-ANNs are actively being used in clinical settings for breast cancer risk assessment and screening)
Shiao et al., 2018 [67]	Machine learning validation techniques	Machine learning validation techniques, such as ensemble methods, generalized regression prediction, elastic net, and leave-one-out cross-validation were used to validate how healthy eating (measured by the HEI index) influences cancer prognosis. (All the mentioned validation techniques are well-established and actively used in clinical research)

## Data Availability

Not applicable.
